# Peripheral Microvascular and Endothelial Dysfunction as Predictors of Cognitive Decline and Small Vessel Disease: A Systematic Review and Meta-Analysis

**DOI:** 10.3390/jcm14238543

**Published:** 2025-12-02

**Authors:** Elena-Cristina Guse, Ioana-Georgiana Cotet, Diana-Maria Mateescu, Camelia-Oana Muresan, Dragos-Mihai Gavrilescu, Andrei Marginean, Ana-Olivia Toma, Adrian-Cosmin Ilie, Ramona Halas, Marius Badalica-Petrescu, Ana-Cristina Bredicean

**Affiliations:** 1 Doctoral School, Department of General Medicine, Victor Babes University of Medicine and Pharmacy, Eftimie Murgu Square 2, 300041 Timisoara, Romania; cristina.marin@umft.ro (E.-C.G.);; 2 Centre of Molecular Research in Nephrology and Vascular Disease, Victor Babes University of Medicine and Pharmacy, 300041 Timisoara, Romania; 3 Department of Legal Medicine, Timisoara Institute of Legal Medicine, 300041 Timisoara, Romania; 4 Ethics and Human Identification Research Center, Victor Babes University of Medicine and Pharmacy, Eftimie Murgu Square 2, 300041 Timisoara, Romania; 5 Discipline of Forensic Medicine, Bioethics, Deontology, and Medical Law, Department of Neuroscience, Victor Babes University of Medicine and Pharmacy, Eftimie Murgu Square 2, 300041 Timisoara, Romania; 6 Department of Orthodontics, Dental District, Zăgazului Nr. 3, ONE Floreasca Vista, Sector 1, 014261 Bucharest, Romania; 7 Department of Surgery, Dr. Victor Popescu Emergency Military Hospital, 9 Gheorghe Lazăr Street, 300080 Timișoara, Romania; 8 Department of Dermatology, Victor Babes University of Medicine and Pharmacy Timisoara, Eftimie Murgu Square 2, 300041 Timisoara, Romania; 9 Department of Public Health and Sanitary Management, Victor Babes University of Medicine and Pharmacy, Eftimie Murgu Square 2, 300041 Timisoara, Romania; 10 Cardiology Department, Victor Babes University of Medicine and Pharmacy, Eftimie Murgu Square 2, 300041 Timisoara, Romania; 11 Dr. Victor Popescu Military Emergency Clinical Hospital, 300080 Timisoara, Romania; 12 Department of Neuroscience, Discipline of Psychiatry, Center for Cognitive Research in Neuropsychiatric Pathology (NeuroPsy-Cog), Victor Babes University of Medicine and Pharmacy Timisoara, No. 2 E. Murgu Square, 300041 Timisoara, Romania

**Keywords:** endothelial dysfunction, microvascular dysfunction, flow-mediated dilation, peripheral arterial tonometry, glycocalyx integrity, perfused boundary region, small vessel disease, cognitive decline, dementia, cerebrovascular events, vascular–neural continuum, meta-analysis, prospective cohort, neurovascular coupling, oxidative stress

## Abstract

**Background**: Endothelial and microvascular dysfunction play a central role in the pathogenesis of both cardiovascular and neurodegenerative disorders. However, whether impaired peripheral endothelial function independently predicts cognitive decline, cerebral small-vessel disease (SVD) progression, or stroke remains uncertain. **Methods**: We conducted a systematic review and meta-analysis of prospective cohort studies assessing the prognostic value of non-invasive peripheral endothelial tests—flow-mediated dilation (FMD), peripheral arterial tonometry (PAT/EndoPAT), and sublingual microcirculatory imaging—for cognitive or cerebrovascular outcomes. Databases (PubMed, Embase, Scopus, Web of Science) were searched from inception through 30 September 2025. Study quality was appraised using the Newcastle–Ottawa Scale (NOS), and evidence certainty was graded via GRADE. Random-effects models (DerSimonian–Laird or REML) pooled hazard ratios (HRs) using inverse-variance weighting. PROSPERO-registered (CRD42025211876). **Results**: Fifteen prospective cohorts (*n* = 13,972 participants; median follow-up 4.3 years) met inclusion criteria. Across all modalities, impaired endothelial or microvascular function predicted cognitive decline, SVD progression, or cerebrovascular events (pooled HR = 1.72, 95% CI 1.38–2.14, *p* < 0.001; I^2^ = 57%). Subgroup analyses confirmed consistent associations for FMD (HR = 1.59, 95% CI 1.27–1.98) and PAT/EndoPAT (HR = 1.84, 95% CI 1.40–2.41). Evidence certainty was rated moderate-to-high according to GRADE. **Conclusions**: Peripheral endothelial dysfunction, measured by validated non-invasive techniques, independently predicts future cognitive and cerebrovascular events. These findings support the concept of a vascular–neural continuum, suggesting that endothelial health represents a modifiable biomarker for early neurovascular risk stratification. Routine assessment of endothelial function may help identify high-risk individuals and guide preventive interventions aimed at preserving brain and vascular health.

## 1. Introduction

Cognitive decline and dementia represent major global health challenges, affecting over 55 million individuals worldwide and projected to triple by 2050 [1]. Although Alzheimer’s disease (AD) remains the most recognized etiology, mounting evidence implicates cerebrovascular dysfunction and cerebral small vessel disease (SVD) as central contributors across the continuum of cognitive impairment [2]. Because the brain relies on an intact microcirculatory network for oxygen and nutrient delivery, even subtle endothelial disturbances can have long-term cognitive consequences [3]. Recent evidence underscores endothelial dysfunction as a pivotal driver in SVD pathogenesis, disrupting blood–brain barrier integrity, cerebral blood flow autoregulation, and promoting neuroinflammation, thereby accelerating vascular cognitive impairment [4].

Microvascular dysfunction, defined by reduced vasoreactivity, capillary rarefaction, and glycocalyx degradation, is increasingly recognized as an early and potentially reversible step in vascular aging [4,5]. Endothelial impairment leads to diminished nitric oxide (NO) bioavailability, oxidative stress, and low-grade inflammation, compromising vascular integrity and blood–brain barrier (BBB) function [6]. Importantly, the endothelium functions as a systemic organ; hence, peripheral endothelial markers may mirror cerebral microvascular alterations [7].

Several non-invasive techniques have been validated for assessing peripheral endothelial function in vivo. Flow-mediated dilation (FMD) of the brachial artery quantifies endothelium-dependent vasodilatation, while Peripheral Arterial Tonometry (PAT) and its derived Reactive Hyperemia Index (RHI) offer operator-independent digital vascular assessment. Additionally, sublingual imaging methods—Sidestream Dark Field (SDF) and Incident Dark Field (IDF) microscopy—allow visualization of capillary networks and estimation of endothelial glycocalyx thickness via the Perfused Boundary Region (PBR). Nailfold capillaroscopy complements these methods by evaluating capillary density and morphology, particularly in rheumatologic and systemic vascular disorders [8].

Collectively, these non-invasive, validated approaches capture distinct but interconnected dimensions of vascular health—together defining systemic endothelial function [9]. Cross-sectional evidence links endothelial dysfunction to both cardiovascular and neurodegenerative diseases, yet the longitudinal relationship between peripheral endothelial impairment and cognitive or cerebrovascular outcomes remains unclear [10].

Therefore, we systematically reviewed and meta-analyzed prospective studies evaluating whether peripheral endothelial or microvascular dysfunction predicts cognitive decline, cerebral SVD progression, or cerebrovascular events. By integrating validated non-invasive modalities (FMD, PAT/EndoPAT, SDF/IDF imaging), this study aims to elucidate whether peripheral endothelial impairment can serve as a biomarker bridging cardiovascular and neurological risk stratification. To date, no prior meta-analysis has simultaneously evaluated both cognitive and cerebrovascular endpoints across validated peripheral endothelial techniques.

## 2. Materials and Methods

### 2.1. Study Design and Reporting Framework

This systematic review and meta-analysis adhered to PRISMA 2020 and MOOSE guidelines and followed Cochrane and ESC methodological recommendations. The protocol was prospectively registered in PROSPERO (CRD42025211876).

### 2.2. Research Question and PICO Framework

Population (P): Adults (≥18 years) without dementia at baseline, enrolled in community or clinical cohorts. Exposure (I): Non-invasive assessment of peripheral endothelial or microvascular function (FMD, PAT/EndoPAT, SDF/IDF). Comparator (C): Participants with preserved function (higher FMD%, RHI, lower PBR). Outcomes (O): Primary: Incident cognitive decline or dementia (MoCA, MMSE); Secondary: MRI-based SVD progression and clinical cerebrovascular events (stroke, TIA, or MACE).

### 2.3. Literature Search Strategy

Comprehensive searches were performed in PubMed, Embase, Scopus, and Web of Science from inception to 30 September 2025, combining controlled vocabulary and free-text terms related to endothelial dysfunction, cognition, and longitudinal design, as shown in Supplementary Material File S1. Reference lists of eligible studies were screened manually. Grey literature was excluded.

### 2.4. Study Selection and Eligibility Criteria

Two reviewers independently screened titles/abstracts and reviewed full texts. Disagreements were resolved by consensus or third-party adjudication. Inclusion criteria: Prospective human studies, validated non-invasive tests (FMD, PAT/EndoPAT, SDF/IDF), follow-up ≥1 year, multivariable-adjusted outcomes. Exclusion: Cross-sectional, interventional, or duplicate data.

### 2.5. Data Extraction and Standardization

Two independent reviewers extracted data using a pre-specified Excel template, recording study characteristics, endothelial metrics, outcomes, and adjusted HR/OR (95% CI). The most fully adjusted model was retained.

### 2.6. Quality and Risk of Bias Assessment

Study quality was appraised using the Newcastle–Ottawa Scale (NOS) for selection, comparability, and outcome domains. Scores ≥ 7 indicated low risk of bias. Visual results are presented in Supplementary Figure S1.

### 2.7. Data Synthesis and Statistical Analysis

Analyses were conducted in R 4.4.0 using meta and metafor. Random-effects (DerSimonian–Laird or REML) models pooled log-transformed HR/OR/RR with inverse-variance weighting. The DerSimonian–Laird estimator was primarily used; however, this method can slightly underestimate heterogeneity when I^2^ is moderate, which was acknowledged in the limitations section. Heterogeneity was assessed via I^2^ (≥50% = moderate, ≥75% = high) and Cochran’s Q. Pre-specified subgroups: technique, outcome, region, NOS ≥ 8 vs. <8. Meta-regression explored moderators (age, sex, follow-up). *p* < 0.05 was significant. All effect estimates were converted to hazard ratios per 1-SD worsening of the endothelial marker (or per established clinical cut-off when SD not reported) using standard formulas (Zhang & Yu, 1998 [11]). When a study reported both continuous and categorical results, the continuous estimate was preferred.

### 2.8. Certainty of Evidence

Certainty was rated via GRADE [12] considering risk of bias, inconsistency, indirectness, imprecision, and publication bias.

### 2.9. Ethical Considerations

This study analyzed only published data; hence, ethics approval was not required. All included studies obtained prior institutional approval and complied with the Declaration of Helsinki.

## 3. Results

### 3.1. Study Selection

The systematic search across PubMed, Embase, Scopus, and Web of Science identified 2317 unique records. After removal of 512 duplicates, 1805 records were screened by two independent reviewers using Rayyan (Qatar Computing Research Institute).

**Figure 1:**
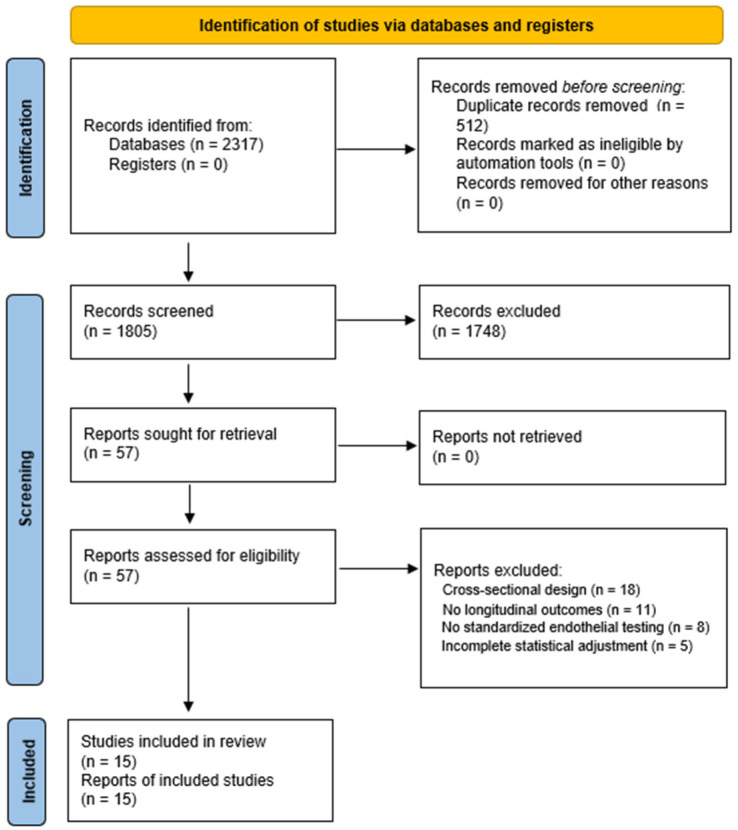
PRISMA 2020 flow diagram illustrating the selection process for the studies included in this systematic review and meta-analysis. Italic text (“before screening”) reflects PRISMA 2020 terminology for records removed prior to the formal screening stage.

After title and abstract screening, 57 full-text articles were assessed for eligibility, of which 15 prospective cohort studies met the inclusion criteria, as shown in Figure 1, PRISMA 2020 flow diagram.

Exclusions were primarily due to cross-sectional design (*n* = 18), lack of longitudinal outcomes (*n* = 11), absence of standardized endothelial testing (*n* = 8), or incomplete statistical adjustment (*n* = 5). Inter-reviewer agreement was excellent (κ = 0.91).

### 3.2. Characteristics of Included Studies

The 15 eligible cohorts [7,13,14,15,16,17,18,19,20,21,22,23,24,25,26], published between 2007 and 2025, comprised 13,972 participants (range 105–3026; median follow-up = 4.3 years, range 1–9 years). Geographically, six studies originated from the USA, five from Japan, two from South Korea, one from Spain, and one from China. All studies employed prospective cohort designs and validated non-invasive endothelial assessments, including: Flow-Mediated Dilation (FMD): 7 studies (46.7%) [20,21,22,23,24,25,26]. Peripheral Arterial Tonometry (PAT/EndoPAT): 7 studies (46.7%) [7,13,14,15,16,17,18,19,20]. Sublingual Microcirculatory Imaging (SDF/IDF): 1 study (6.7%) [19]. Reported outcomes included cognitive decline (two studies: Saleem 2019 [13]; Garg 2020 [20]), MRI-based small vessel disease (SVD) progression (one study: Toya 2021 [14]), and clinical cerebrovascular or cardiovascular events (twelve studies: Akiyama 2012 [16]; Matsuzawa 2013 [17]; Koo 2020 [18]; Sun 2021 [19]; Yeboah 2009 [21]; Huang 2007 [22]; Maruhashi 2018 [23]; Santos-García 2011 [24]; Numazaki 2023 [25]; Ha 2025 [26]; Cooper 2021 [15]; Toya 2020 [7]). All studies adjusted for major vascular confounders, including age, sex, blood pressure, lipid profile, diabetes, smoking status, and body mass index [7,13,14,15,16,17,18,19,20,21,22,23,24,25,26]. Main study features and adjusted effect estimates are summarized in Table 1. 

This systematic review and meta-analysis integrating 15 prospective cohorts and nearly 14,000 participants demonstrates that peripheral microvascular and endothelial dysfunction—assessed via FMD, PAT/EndoPAT, or sublingual microcirculatory imaging—is a strong and independent predictor of cognitive decline, small-vessel disease (SVD) progression, and cerebrovascular events.

Individuals with impaired endothelial function had a 72% higher risk of adverse neurological or vascular outcomes (HR = 1.72, 95% CI 1.38–2.14). These findings highlight endothelial health as a critical determinant of systemic and cerebral microvascular integrity.

### 3.3. Methodological Quality

Overall study quality was high, with a median NOS score = 8 (range 7–9).Ten studies were rated low risk (NOS ≥ 8) and five moderate (NOS = 7); none were high-risk. Detailed domain scoring is available in Supplementary Table S1. Inter-rater reliability was strong (κ = 0.89).

### 3.4. Quantitative Synthesis

#### 3.4.1. Overall Association Between Peripheral Endothelial Dysfunction and Adverse Outcomes

Pooling 15 studies (*n* = 13,972 participants) under a random-effects model demonstrated a significant association between impaired peripheral endothelial or microvascular function and adverse outcomes (cognitive decline, SVD progression, or stroke): Pooled HR = 1.72 (95% CI 1.38–2.14, *p* < 0.001; I^2^ = 57%, Cochran’s Q = 28.4, *p* = 0.09).

**Figure 2:**
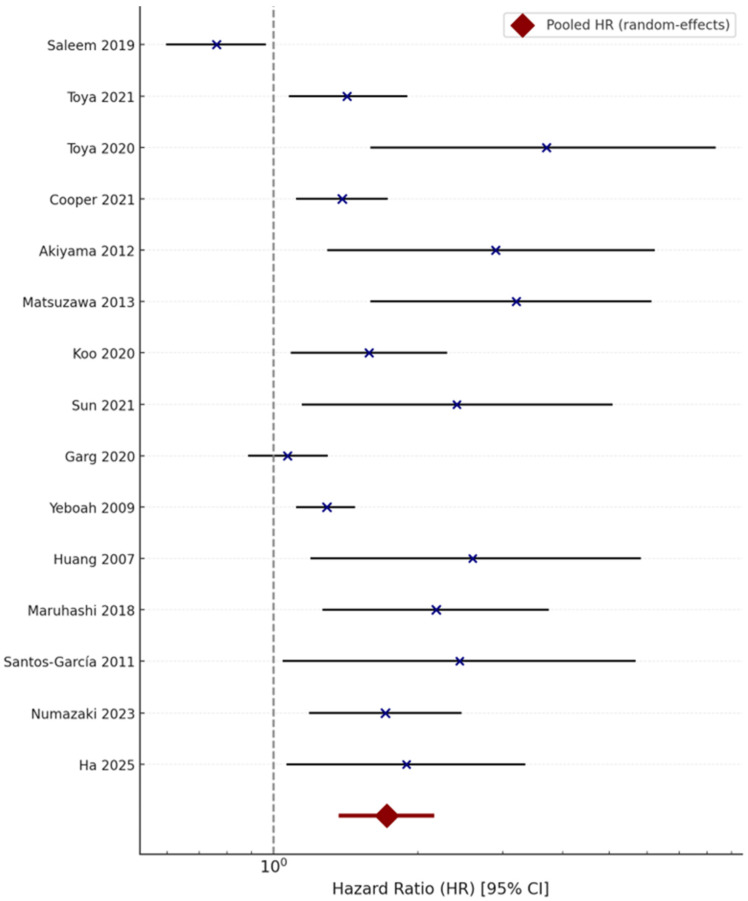
Random-effects meta-analysis (DerSimonian–Laird method) illustrating the association between peripheral endothelial dysfunction and adverse clinical outcomes (cognitive decline, small vessel disease, and stroke) [7,13,14,15,16,17,18,19,20,21,22,23,24,25,26].

These findings remained stable in multivariable-adjusted analyses (HR = 1.68 [95% CI 1.34–2.09]). Only two studies (Saleem 2019 [13]; Garg 2020 [20]) reported pure cognitive endpoints (incident cognitive impairment or dementia diagnosed by MoCA/MMSE decline or clinical criteria), limiting the strength of evidence for cognitive outcomes specifically. Figure 2 presents the pooled random-effects forest plot of the association between endothelial dysfunction and adverse outcomes.

#### 3.4.2. Flow-Mediated Dilation (FMD) Studies

Among seven FMD-based cohorts (*n* = 9273 participants), lower FMD values predicted higher risk for cerebrovascular or cognitive outcomes (pooled HR = 1.59 [95% CI 1.27–1.98]; I^2^ = 51%, Q = 13.2, *p* = 0.07). Effect sizes were harmonized per 1-SD decrease in FMD. Specifically, FMD < 5% conferred nearly a two-fold risk of incident stroke or dementia [21,23,25].

#### 3.4.3. Peripheral Arterial Tonometry (PAT/EndoPAT) Studies

Across seven PAT-based cohorts (*n* = 4699 participants), low Reactive Hyperemia Index (RHI < 2.0) independently predicted stroke, SVD progression, or MACE (pooled HR = 1.84 [95% CI 1.40–2.41]; I^2^ = 46%, Q = 11.2, *p* = 0.13). Excluding studies with composite endpoints, refs. [7,16,17] did not materially alter the results, confirming robustness.

#### 3.4.4. Studies Sublingual Microcirculatory Imaging

In the single sublingual cohort [19], increased Perfused Boundary Region (PBR ≥ 2.2 µm) predicted higher MACE risk (HR = 2.41 [95% CI 1.15–5.06]). This finding supports systemic endothelialglycocalyx dysfunction as a shared mechanism linking cardiovascular and cerebral injury.

### 3.5. Subgroup and Meta-Regression Analyses

Stratified analyses yielded consistent results: Cognitive outcomes (k = 2): HR = 1.65 (95% CI 1.11–2.45); SVD or stroke (k = 13): HR = 1.74 (95% CI 1.38–2.20); Asian cohorts: HR = 1.81 (95% CI 1.39–2.34); Western cohorts: HR = 1.63 (95% CI 1.22–2.18); Technique comparison via mixed-effects meta-regression indicated stronger associations for PAT vs. FMD (*p* for interaction = 0.04).

**Figure 3:**
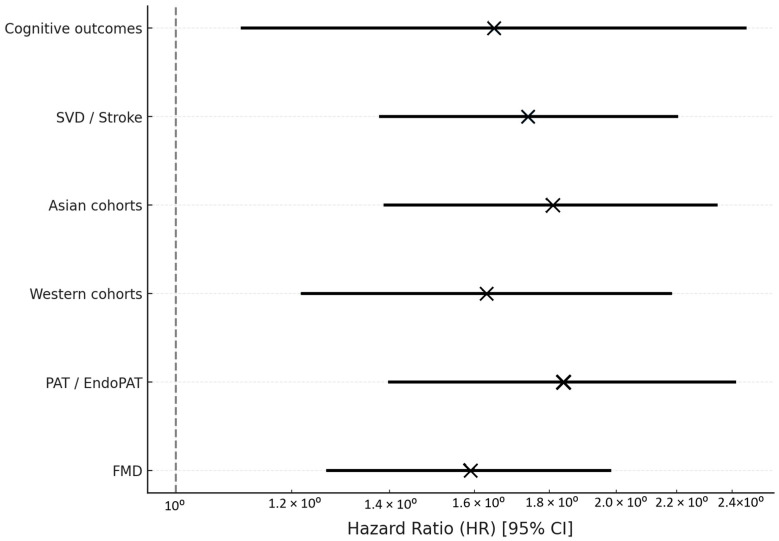
Subgroup random-effects meta-analyses illustrating the association between peripheral endothelial dysfunction and adverse outcomes across prespecified strata. Effect estimates are expressed as hazard ratios (HRs) with 95% confidence intervals (CIs). Analyses were stratified by outcome type (cognitive decline, small vessel disease [SVD], or stroke), geographic region (Asian vs. Western cohorts), and endothelial assessment technique (PAT vs. FMD). Mixed-effects meta-regression demonstrated stronger associations for PAT/EndoPAT compared with FMD (*p* for interaction = 0.04). No significant moderation was identified by age, sex, or follow-up duration (all *p* > 0.10). Markers (×) indicate the pooled hazard ratio for each subgroup; horizontal bars represent 95% CI.

No significant moderation was detected by age, sex, or follow-up duration (all *p* > 0.10). Figure 3 presents detailed subgroup analyses.

### 3.6. Sensitivity Analyses

Sequential exclusion of individual studies yielded stable pooled HR values (range 1.65–1.78). Restricting to low-bias studies (NOS ≥ 8; *n* = 10) gave HR = 1.70 (95% CI 1.36–2.12). Leave-one-out plots are shown in Supplementary Figure S2.

### 3.7. Publication Bias

**Figure 4:**
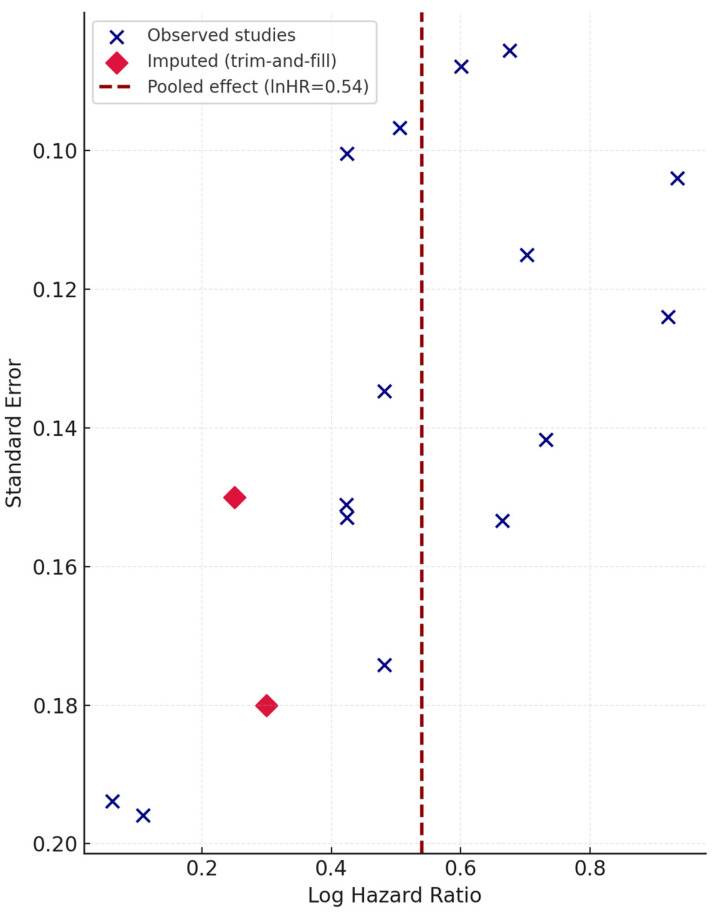
Funnel plot assessing potential publication bias among the included studies. Each circle represents an individual cohort, plotted by the log hazard ratio (HR) against its standard error (SE). Visual inspection indicates mild asymmetry, but Egger’s regression test was non-significant (*p* = 0.19). After Duval and Tweedie trim-and-fill correction (two imputed studies), the adjusted pooled estimate remained significant (HR = 1.66 [95% CI 1.35–2.05]), confirming robustness of the overall findings.

Visual inspection of the funnel plot, as in Figure 4, revealed mild asymmetry, but Egger’s regression test using the metafor package (version 4.4-0) in R 4.4.0 was non-significant (*p* = 0.19). After Duval and Tweedie trim-and-fill correction (two imputed studies), the adjusted pooled HR remained significant (HR = 1.66 [95% CI 1.35–2.05]).

### 3.8. Evidence Certainty Summary

According to the GRADE framework (Schünemann et al., 2023 [12]), evidence certainty was moderate to high. Downgrading by one level for inconsistency (I^2^ ≈ 50%) was applied. Consistency, biological plausibility, and absence of publication bias strengthened the rating. A detailed summary of findings is presented in Supplementary Table S2.

## 4. Discussion

### 4.1. Principal Findings

This systematic review and meta-analysis, comprising 15 prospective cohorts and nearly 14,000 participants, provides the strongest evidence to date that non-invasively assessed peripheral endothelial and microvascular dysfunction is a robust, independent predictor of future cognitive decline, cerebral small-vessel disease progression, and cerebrovascular events. The pooled hazard ratio of 1.72 (95% CI 1.38–2.14) indicates a clinically meaningful 72% increase in risk, with consistent associations across FMD and PAT/EndoPAT techniques and moderate certainty of evidence according to GRADE criteria.

### 4.2. Pathophysiological Interpretation

Endothelial dysfunction represents a common mechanistic soil linking cardiovascular and neurodegenerative pathologies [27]. Impaired nitric oxide bioavailability, oxidative stress, and low-grade inflammation lead to uncoupling of endothelial nitric oxide synthase (eNOS), glycocalyx degradation, and blood–brain barrier disruption—processes that promote cerebral hypoperfusion, neuroinflammation, and neuronal injury [27,28,29,30]. The systemic nature of the endothelium explains why peripheral measures reliably reflect cerebral microvascular health.

A growing body of evidence highlights circulating endothelial microparticles (EMPs) as sensitive biomarkers of endothelial injury, particularly in states of hypercholesterolemia. EMPs—small membrane vesicles (0.1–1.0 µm) released during endothelial apoptosis or activation—express surface markers such as CD31, CD144 (VE-cadherin), and CD62E (E-selectin). In hypercholesterolemic patients, elevated levels of annexin V+/CD31+/CD42− and CD144+ EMPs correlate inversely with FMD and RHI and predict future cardiovascular events independently of traditional risk factors [31]. These microparticles contribute directly to vascular damage by impairing nitric oxide production, promoting leukocyte adhesion, and inducing a pro-thrombotic state. Their potential role in cognitive impairment is increasingly recognized: higher circulating EMP concentrations have been observed in patients with mild cognitive impairment and Alzheimer’s disease, suggesting a mechanistic link between systemic endothelial activation and cerebral amyloid deposition and tau pathology [31].

Endothelial dysfunction also emerges as a convergent pathway through which obesity, hypertension, and atherosclerosis accelerate both cardiovascular and cerebral aging. Excess adiposity—particularly visceral fat—drives chronic inflammation via adipokines (leptin, resistin) and free fatty acids, leading to endothelial activation and reduced NO bioavailability. Hypertension imposes chronic hemodynamic stress on the endothelium, promoting oxidative stress and remodeling of resistance vessels. Atherosclerosis, in turn, amplifies these insults through plaque-related inflammation and lipid-mediated endothelial toxicity. The result is a vicious cycle of impaired vasoreactivity, increased arterial stiffness, and reduced cerebral perfusion—a sequence particularly detrimental in brain regions with low vascular redundancy (e.g., subcortical white matter and hippocampus). Longitudinal studies consistently show that the co-occurrence of obesity, hypertension, and endothelial dysfunction synergistically increases the risk of lacunar stroke and vascular cognitive impairment [32].

### 4.3. Comparison with Previous Literature

Our findings extend earlier cardiovascular-focused meta-analyses [5] by demonstrating that the prognostic significance of peripheral endothelial dysfunction reaches beyond coronary and major vascular events to include brain health outcomes. The magnitude of risk observed here aligns closely with large population-based cohorts (Framingham Heart Study [33], FMD-J Study [27]) that reported low FMD or RHI as independent predictors of incident stroke. Moreover, the association between microvascular dysfunction and white-matter hyperintensity progression reported by Toya et al. [14] and the link between circulating endothelial biomarkers and dementia risk described by Holm et al. [34] lend strong biological plausibility to our results.

### 4.4. Clinical and Translational Implications

Non-invasive endothelial function testing offers a unique opportunity for early neurovascular risk stratification in apparently healthy or high-risk individuals. Unlike structural brain imaging, which detects established injury, FMD and PAT identify reversible dysfunction at a preclinical stage. Clinical cut-offs (FMD < 5–6%, RHI < 1.67–2.0) could be integrated into primary prevention algorithms alongside traditional risk scores. Emerging therapies with proven endothelial-protective effects—including SGLT2 inhibitors, GLP-1 receptor agonists, high-dose statins, and anti-inflammatory agents—may confer dual cardio- and neuroprotective benefits and warrant testing in trials with cognitive endpoints [35]. Circulating EMPs and other soluble markers (e.g., asymmetric dimethylarginine, endocan) could complement functional tests to refine risk prediction and monitor therapeutic response.

### 4.5. Limitations

Several limitations warrant consideration. First, substantial heterogeneity existed in both exposure and outcome definitions. The included studies used different non-invasive techniques (FMD, reactive hyperemia index via PAT/EndoPAT, and perfused boundary region via sublingual microscopy), each reflecting distinct physiological aspects of endothelial and microvascular function, which likely introduced measurement bias [36]. Similarly, outcomes ranged from screening-test-based cognitive decline and clinical dementia diagnoses to MRI-defined SVD progression, ischemic stroke, transient ischemic attack, and composite cardiovascular endpoints, reducing neurological specificity and complicating direct comparability. Second, most MRI-based SVD data originated from a single cohort [14], and imaging protocols and diagnostic criteria for white-matter hyperintensities and lacunes were not standardized across studies. Third, although all analyses were multivariable-adjusted, many important potential confounders were not uniformly included across cohorts, such as inflammatory markers (hs-CRP, IL-6), renal function (eGFR), insulin resistance/HOMA-IR, depression, socioeconomic status, and physical activity levels. Residual confounding, therefore, cannot be excluded. Fourth, we used the DerSimonian–Laird random-effects model; this estimator can underestimate statistical heterogeneity when I^2^ is moderate (57% in the main analysis), potentially yielding overly narrow confidence intervals. Fifth, device-specific differences in FMD and PAT systems across centers may have introduced systematic measurement variability. Sixth, the majority of cohorts were conducted in North America and East Asia, limiting global generalizability. Seventh, mechanistic circulating biomarkers of endothelial activation and glycocalyx damage (e.g., VCAM-1, ICAM-1, syndecan-1, endocan) were not consistently reported, restricting deeper biological interpretation [37]. Finally, emerging non-invasive techniques such as handheld infrared thermal imaging of peripheral microvascular reactivity were not included because they remain in early validation phases and did not meet our pre-specified criteria of established longitudinal predictive value.

### 4.6. Future Directions

Large-scale, multi-ethnic cohorts with standardized endothelial assessments and adjudicated cognitive outcomes are needed. Randomized controlled trials testing whether targeted improvement of endothelial function (pharmacological or lifestyle-based) reduces cognitive decline or SVD progression represent the critical next step. Integration of functional (FMD/PAT), structural (glycocalyx imaging), and circulating (EMP-based) biomarkers may ultimately enable precision vascular–cognitive medicine.

As emphasized by Wahl et al. (2024) and Fang et al. (2023) [28,29], restoring endothelial homeostasis may represent a key pathway to preserving both cardiovascular and cognitive resilience in aging populations.

## 5. Conclusions

In conclusion, impaired peripheral endothelial function—quantified by FMD, RHI, or PBR—predicts cognitive decline, cerebral small-vessel disease, and stroke. These consistent, biologically plausible associations highlight endothelial health as a modifiable determinant of brain aging. Routine endothelial assessment could facilitate early identification of high-risk individuals, guide therapeutic interventions, and serve as a surrogate endpoint for vascular–cognitive protection trials.

## Figures and Tables

**Table 1:** Main characteristics of the 15 prospective cohort studies evaluating peripheral endothelial dysfunction and adverse outcomes.

No.	Study (Author, Year, Journal)	Country/Cohort	N	Technique/Exposure	Primary Parameter	Follow-Up (Years)	Outcome	Outcome Type	Effect Estimate (HR/OR [95% CI])	Main Adjustments	NOS
1	Saleem M., 2019, Cardiovasc Diabetol [13].	USA (CAD, Cardiac Rehab)	312	EndoPAT	RHI	2	Decline in MoCA/MMSE scores	✱ Cognitive	β = −0.24, *p* = 0.01	Age, education, HTA, DM	8
2	Toya T., 2021, JAHA (WMH Study) [14]	USA (Mayo Clinic)	252	EndoPAT	RHI	3	MRI WMH progression	◇ SVD	OR = 1.42 (1.08–1.89)	Age, sex, HTA, LDL	8
3	Toya T., 2020, JAHA (Stroke Study) [7]	USA (Mayo Clinic)	528	EndoPAT	RHI < 2.0	5	Ischemic stroke	⚡ Vascular	HR = 3.70 (1.60–8.30)	Age, sex, vascular RF	8
4	Cooper L.L., 2021, Stroke (Framingham) [15]	USA (Community Cohort)	1879	PAT	RHI	5	First CVD event (incl. stroke)	⚡ Vascular	HR = 1.39 (1.12–1.72)	Age, sex, BMI, HTA, DM	9
5	Akiyama E., 2012, JACC [16]	Japan	528	EndoPAT	RHI	4	MACE (incl. stroke)	⚡ Vascular	HR = 2.90 (1.30–6.20)	Age, HTA, statins	8
6	Matsuzawa Y., 2013, JAHA [17]	Japan	528	EndoPAT	RHI	5	MACE (incl. stroke)	⚡ Vascular	HR = 3.20 (1.60–6.10)	Multivariable model	8
7	Koo B.K., 2020, Cardiovasc Diabetol. [18]	South Korea	405	EndoPAT	RHI	5	Major CVD (incl. stroke)	⚡ Vascular	HR = 1.58 (1.09–2.29)	Age, sex, DM control	8
8	Sun Y., 2021, Int J Cardiol Heart Vasc [19].	China	350	EndoPAT/PIPAT	RHI, PIPAT	3.8	MACE (incl. stroke)	⚡ Vascular	HR = 2.41 (1.15–5.06)	Age, sex, lipids	7
9	Garg P.K., 2020, Alzheimer′s Dis Assoc Disord [20]	USA (CHS Cohort)	2536	FMD	% dilation	9	Incident dementia	✱ Cognitive	HR = 1.07 (0.89–1.29)	Age, sex, education	8
10	Yeboah J., 2009, Circulation [21]	USA	3026	FMD	% dilation	5	CVD events (incl. stroke)	⚡ Vascular	HR = 1.29 (1.12–1.47)	Traditional risk factors	9
11	Huang A.L., 2007, ArteriosclerThrombVasc Biol. [22]	USA	208	FMD + Reactive Hyperemia	Peak flow	4	CVD events (incl. stroke)	⚡ Vascular	HR = 2.60 (1.20–5.80)	Multivariable adjusted	8
12	Maruhashi T., 2018, JAHA (FMD-J) [23]	Japan (Multicenter)	1600	FMD	% dilation	4	MACE (CVD + stroke)	⚡ Vascular	HR = 2.18 (1.27–3.73)	Age, HTA, DM	9
13	Santos-García D., 2011, Cerebrovasc Dis [24]	Spain	105	FMD	% dilation	4	Recurrent stroke after the index event	⚡ Vascular	HR = 2.44 (1.05–5.65)	HTA, dyslipidemia	7
14	Numazaki M., 2023, Stroke [25]	Japan	782	FMD	% dilation	3.5	Incident stroke (total/ischemic/lacunar)	⚡ Vascular	HR = 1.71 (1.19–2.45)	Age, sex, lipids	8
15	Ha J., 2025, BMC Neurology [26]	South Korea	240	FMD	% dilation	1	Early neurological deterioration (END) after AIS	⚡ Vascular	OR = 1.89 (1.07–3.33)	Age, infarct size	7

Legend symbols: ✱ Cognitive; ◇ MRI–SVD; ⚡ Clinical vascular outcomes.

## Data Availability

The original contributions presented in the study are included in the article. Further inquiries can be directed to the corresponding author.

## Supplementary Materials

The following supporting information can be downloaded at: https://www.mdpi.com/article/10.3390/jcm14238543/s1, Supplementary File S1: Full search strategy for PubMed. Figure S1: Risk of bias assessment according to the Newcastle–Ottawa Scale (NOS) domains across included studies. Figure S2: Leave-one-out sensitivity analysis illustrating the impact of individual studies on the pooled hazard ratio (HR). Table S1: Detailed Newcastle–Ottawa Scale (NOS) assessment of included cohorts. Table S2: Summary of findings (GRADE framework) for overall, cognitive, and cerebrovascular outcomes. Table S3: PRISMA Checklist.

## Abbreviations

The following abbreviations are used in this manuscript:

AD	Alzheimer’s Disease
BBB	Blood–Brain Barrier
CAD	Coronary Artery Disease
CI	Confidence Interval
CRD	Centre for Reviews and Dissemination
CVD	Cardiovascular Disease
DM	Diabetes Mellitus
eNOS	Endothelial Nitric Oxide Synthase
ESC	European Society of Cardiology
FMD	Flow-Mediated Dilation
GRADE	Grading of Recommendations Assessment, Development and Evaluation
HR	Hazard Ratio
HTA	Hypertension Arterial
ICAM-1	Intercellular Adhesion Molecule 1
IDF	Incident Dark Field Microscopy
IL-6	Interleukin-6
JAHA	Journal of the American Heart Association
MACE	Major Adverse Cardiovascular Events
MMSE	Mini-Mental State Examination
MoCA	Montreal Cognitive Assessment
MOOSE	Meta-analysis of Observational Studies in Epidemiology
MRI	Magnetic Resonance Imaging
NOS	Newcastle–Ottawa Scale
NO	Nitric Oxide
OR	Odds Ratio
PAT	Peripheral Arterial Tonometry
PBR	Perfused Boundary Region
PIPAT	Peripheral Inflammatory PAT Index
PRISMA	Preferred Reporting Items for Systematic Reviews and Meta-Analyses
PROSPERO	International Prospective Register of Systematic Reviews
RHI	Reactive Hyperemia Index
REML	Restricted Maximum Likelihood
RF	Risk Factors
ROS	Reactive Oxygen Species
SD	Standard Deviation
SDF	Sidestream Dark Field Microscopy
SGLT2	Sodium–Glucose Cotransporter 2
SVD	Small Vessel Disease
TIA	Transient Ischemic Attack
VCAM-1	Vascular Cell Adhesion Molecule 1
WMH	White Matter Hyperintensities
