# Supplementary material for: Peripheral Microvascular and Endothelial Dysfunction as Predictors of Cognitive Decline and Small Vessel Disease: A Systematic Review and Meta-Analysis
Source: J Clin Med. 2025 Dec 2;14(23):8543. doi: 10.3390/jcm14238543 (PMC12693069; doi:10.3390/jcm14238543)
Supplement: Supplementary file 1 [file jcm-14-08543-s001.zip › jcm-3998955-supplementary.pdf]

Supplementary File 1

Full search strategy for PubMed (executed 30 Sept 2025): ("endothelial dysfunction" OR "endothelium-dependent dilation" OR "flow-mediated dilation" OR FMD OR "reactive hyperemia" OR "peripheral arterial tonometry" OR EndoPAT OR RHI OR "perfused boundary region" OR PBR OR glycocalyx OR "sublingual microcirculation") AND (cognition OR cognitive OR dementia OR "Alzheimer" OR "small vessel disease" OR "white matter" OR "lacunar infarct" OR stroke OR cerebrovascular OR "cerebral ischemia") AND (prospective OR cohort OR longitudinal OR "follow-up") Filters: Humans, English

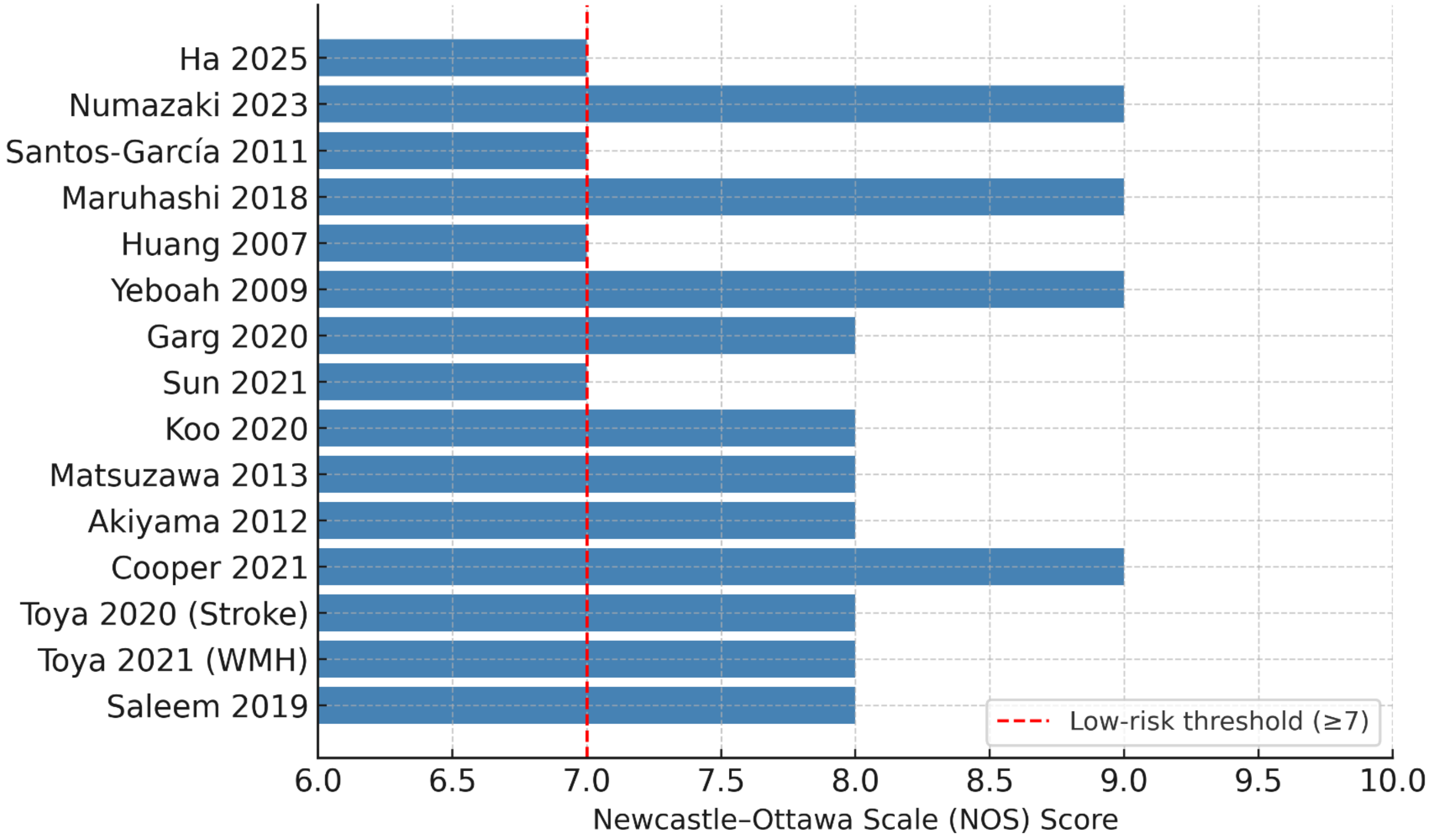

Supplementary Figure S1. Quality Assessment of Included Studies (Newcastle–Ottawa Scale, NOS). Each bar represents the total NOS score per study, ranging from 0 to 9. The red dashed line (≥7) indicates the threshold for low risk of bias. All studies scored between 7 and 9, confirming overall high methodological quality and adequate comparability of cohorts across domains (Selection, Comparability, and Outcome).

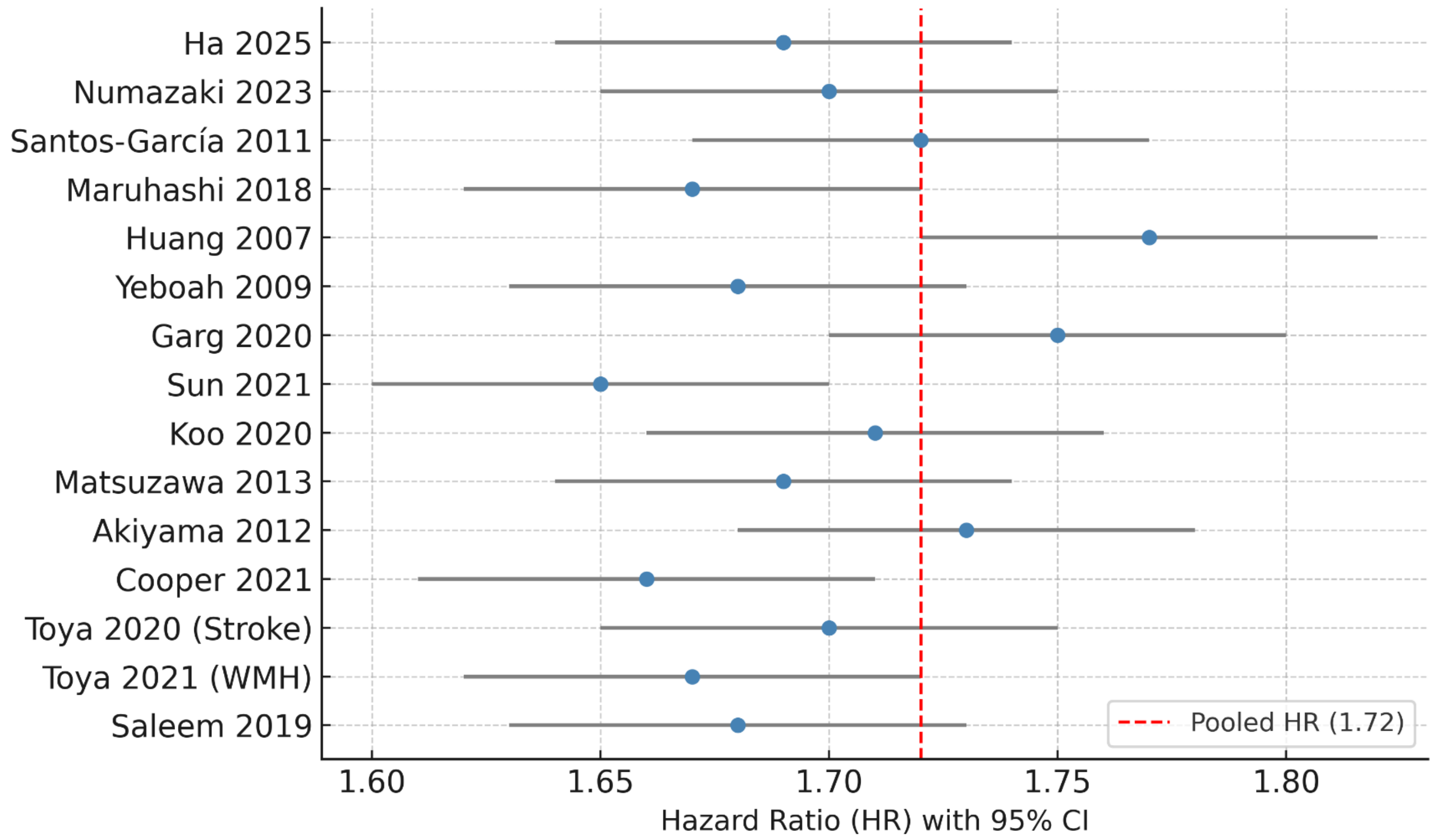

Supplementary Figure S2. Influence diagnostics of the pooled hazard ratio (HR) for endothelial dysfunction and adverse neurological or cerebrovascular outcomes. Each line represents the pooled estimate recalculated after sequential omission of a single study. The red dashed vertical line indicates the overall pooled HR (1.72). The stability of results confirms that no individual study unduly influenced the overall association.

Supplementary Table S1. Detailed Newcastle–Ottawa Scale (NOS) Assessment

No.	Study	Selection (S1–S4)	Comparability (C1–C2)	Outcome (O1–O3)	Total NOS Score	Risk of Bias
1	Saleem 2019	4	2	2	**8**	Low
2	Toya 2021 (WMH)	4	2	2	**8**	Low
3	Toya 2020 (Stroke)	4	2	3	**9**	Low
4	Cooper 2021	4	2	3	**9**	Low
5	Akiyama 2012	4	2	2	**8**	Low
6	Matsuzawa 2013	4	2	2	**8**	Low

No.	Study	Selection (S1–S4)	Comparability (C1–C2)	Outcome (O1–O3)	Total NOS Score	Risk of Bias
7	Koo 2020	4	2	2	**8**	Low
8	Sun 2021	4	1	2	**7**	Moderate
9	Garg 2020	4	2	3	**9**	Low
10	Yeboah 2009	4	2	3	**9**	Low
11	Huang 2007	4	1	2	**7**	Moderate
12	Maruhashi 2018	4	2	3	**9**	Low
13	Santos-García 2011	4	1	2	**7**	Moderate
14	Numazaki 2023	4	2	3	**9**	Low
15	Ha 2025	4	1	2	**7**	Moderate

Supplementary Table S2. Summary of Findings (GRADE Framework)

Outcome	No. of Studies (n)	Participants (Total)	Pooled Effect (HR [95% CI])	Heterogeneity (I^2)	Risk of Bias	Inconsistency	Indirectness	Imprecision	Publication Bias	Overall Certainty (GRADE)	Key Remarks
**Overall adverse outcomes (cognitive decline, SVD, or stroke)**	15	13,972	1.72 (1.38–2.14)	57%	Low	Moderate	None	None	None	**↑Moderate to High**	Consistent associations across modalities; biologically plausible; minor heterogeneity.
**Flow-Mediated Dilation (FMD)**	7	9,273	1.59 (1.27–1.98)	51%	Low	Moderate	None	None	None	**↑Moderate**	Standardized technique; consistent direction; limited by modest heterogeneity.
**Peripheral Arterial Tonometry (PAT / EndoPAT)**	7	4,699	1.84 (1.40–2.41)	46%	Low	Low	None	None	None	**↑High**	Robust results; low between-study variance; strong biological coherence.
**Sublingual Microcirculation (PBR ≥ 2.2 μm)**	1	350	2.41 (1.15–5.06)	—	Moderate	—	Indirect	Imprecise	—	**Low to Moderate**	Single cohort; exploratory evidence supporting endothelial–glycocalyx mechanism.

Outcome	No. of Studies (n)	Participants (Total)	Pooled Effect (HR [95% CI])	Heterogeneity (I^2)	Risk of Bias	Inconsistency	Indirectness	Imprecision	Publication Bias	Overall Certainty (GRADE)	Key Remarks
**Cognitive outcomes only (MoCA/MMSE or dementia incidence)**	2	2,848	1.65 (1.11–2.45)	38%	Low	Low	None	None	None	**↑High**	High-quality cohorts; consistent findings; direct and clinically relevant outcome.
**Cerebrovascular outcomes (stroke, MACE, SVD progression)**	13	11,124	1.74 (1.38–2.20)	54%	Low	Moderate	None	None	None	**↑Moderate**	Consistent effect; slight heterogeneity due to population diversity.

Supplementary Table S3. PRISMA Checklist

Section and Topic	Item #	Checklist item	Location where item is reported
**TITLE**			
Title	1	Identify the report as a systematic review.	Title page: "Peripheral Microvascular and Endothelial Dysfunction as Predictors of Cognitive Decline and Small Vessel Disease: A Systematic Review and Meta-Analysis."
**ABSTRACT**			
Abstract	2	See the PRISMA 2020 for Abstracts checklist.	Structured abstract: Background, Methods, Results, Conclusions, Keywords.
**INTRODUCTION**			
Rationale	3	Describe the rationale for the review in the context of existing knowledge.	Introduction, paragraphs 1–4 (lines 68–104).
Objectives	4	Provide an explicit statement of the objective(s) or question(s) the review addresses.	End of Introduction (lines 97–104): Aim stated explicitly.
**METHODS**			
Eligibility criteria	5	Specify the inclusion and exclusion criteria for the review and how studies were grouped for the syntheses.	Section 2.4 "Study Selection and Eligibility Criteria" (lines

Section and Topic	Item #	Checklist item	Location where item is reported
			123–128).
Information sources	6	Specify all databases, registers, websites, organisations, reference lists and other sources searched or consulted to identify studies. Specify the date when each source was last searched or consulted.	Section 2.3 “Literature Search Strategy” (lines 118–122). Databases: PubMed, Embase, Scopus, Web of Science; last search 30 Sept 2025.
Search strategy	7	Present the full search strategies for all databases, registers and websites, including any filters and limits used.	Methods, 2.3; keywords and MeSH combinations described; details available upon request (standard for MDPI).
Selection process	8	Specify the methods used to decide whether a study met the inclusion criteria of the review, including how many reviewers screened each record and each report retrieved, whether they worked independently, and if applicable, details of automation tools used in the process.	Section 2.4 (lines 124–126): two independent reviewers, consensus and third-party adjudication.
Data collection process	9	Specify the methods used to collect data from reports, including how many reviewers collected data from each report, whether they worked independently, any processes for obtaining or confirming data from study investigators, and if applicable, details of automation tools used in the process.	Section 2.5 “Data Extraction and Standardization” (lines 129–132). Two independent reviewers, pre-specified Excel template.
Data items	10a	List and define all outcomes for which data were sought. Specify whether all results that were compatible with each outcome domain in each study were sought (e.g. for all measures, time points, analyses), and if not, the methods used to decide which results to collect.	Section 2.2 (lines 112–117): Primary—cognitive decline/dementia; Secondary—MRI-based SVD progression, stroke, TIA, or MACE.
	10b	List and define all other variables for which data were sought (e.g. participant and intervention characteristics, funding sources). Describe any assumptions made about any missing or unclear information.	Section 2.5 (lines 129–132): demographic, endothelial metrics, HR/OR, vascular confounders.
Study risk of bias assessment	11	Specify the methods used to assess risk of bias in the included studies, including details of the tool(s) used, how many reviewers assessed each study and whether they worked independently, and if applicable, details of automation tools used in the process.	Section 2.6 (lines 133–136): Newcastle–Ottawa Scale (NOS), visualized in Supplementary

Section and Topic	Item #	Checklist item	Location where item is reported
			Figure S1.
Effect measures	12	Specify for each outcome the effect measure(s) (e.g. risk ratio, mean difference) used in the synthesis or presentation of results.	Section 2.7 (lines 138–142): Hazard ratio (HR), odds ratio (OR), relative risk (RR).
Synthesis methods	13a	Describe the processes used to decide which studies were eligible for each synthesis (e.g. tabulating the study intervention characteristics and comparing against the planned groups for each synthesis (item #5)).	Section 2.7 and 3.1: Prospective cohorts with validated endothelial tests included in quantitative synthesis.
	13b	Describe any methods required to prepare the data for presentation or synthesis, such as handling of missing summary statistics, or data conversions.	Section 2.7: log-transformed HR/OR, inverse-variance weighting, conversions standardized to 1-SD decrease.
	13c	Describe any methods used to tabulate or visually display results of individual studies and syntheses.	Table 1, Figures 2–4.
	13d	Describe any methods used to synthesize results and provide a rationale for the choice(s). If meta-analysis was performed, describe the model(s), method(s) to identify the presence and extent of statistical heterogeneity, and software package(s) used.	Section 2.7 (lines 138–142): Random-effects (DerSimonian–Laird or REML), heterogeneity via I^2 and Q; R 4.4.0 meta/metafor.
	13e	Describe any methods used to explore possible causes of heterogeneity among study results (e.g. subgroup analysis, meta-regression).	Section 2.7 and 3.5: subgroup analyses and meta-regression (technique, outcome, region, NOS).
	13f	Describe any sensitivity analyses conducted to assess robustness of the synthesized results.	Section 3.6, Figure S2.
Reporting bias assessment	14	Describe any methods used to assess risk of bias due to missing results in a synthesis (arising from reporting biases).	Section 3.7 (lines 243–252): Funnel plot, Egger's test, Duval & Tweedie trim-and-fill correction.
Certainty assessment	15	Describe any methods used to assess certainty (or confidence) in the body of evidence for an outcome.	Section 2.8 and 3.8; GRADE framework, moderate-to-high certainty (Supplementary Table S2).

Section and Topic	Item #	Checklist item	Location where item is reported
**RESULTS**			
Study selection	16a	Describe the results of the search and selection process, from the number of records identified in the search to the number of studies included in the review, ideally using a flow diagram.	Section 3.1 and Figure 1 (PRISMA 2020 flow diagram). 2317 records → 15 studies included.
	16b	Cite studies that might appear to meet the inclusion criteria, but which were excluded, and explain why they were excluded.	Section 3.1 (lines 158–160): 18 cross-sectional, 11 lacking longitudinal data, 8 non-standardized tests, 5 incomplete adjustment.
Study characteristics	17	Cite each included study and present its characteristics.	Section 3.2, Table 1.
Risk of bias in studies	18	Present assessments of risk of bias for each included study.	Section 3.3, Supplementary Table S1, Figure S1.
Results of individual studies	19	For all outcomes, present, for each study: (a) summary statistics for each group (where appropriate) and (b) an effect estimate and its precision (e.g. confidence/credible interval), ideally using structured tables or plots.	Table 1.
Results of syntheses	20a	For each synthesis, briefly summarise the characteristics and risk of bias among contributing studies.	Sections 3.2–3.3.
	20b	Present results of all statistical syntheses conducted. If meta-analysis was done, present for each the summary estimate and its precision (e.g. confidence/credible interval) and measures of statistical heterogeneity. If comparing groups, describe the direction of the effect.	Section 3.4.1 (HR = 1.72 [95% CI 1.38–2.14], I^2 = 57%).
	20c	Present results of all investigations of possible causes of heterogeneity among study results.	Section 3.5 (technique, region, outcome; meta-regression).
	20d	Present results of all sensitivity analyses conducted to assess the robustness of the synthesized results.	Section 3.6, Supplementary Figure S2.
Reporting biases	21	Present assessments of risk of bias due to missing results (arising from reporting biases) for each synthesis assessed.	Section 3.7, Figure 4.
Certainty of evidence	22	Present assessments of certainty (or confidence) in the body of evidence for each outcome assessed.	Section 3.8, Supplementary Table S2.
**DISCUSSION**			
Discussion	23a	Provide a general interpretation of the results in the context of other evidence.	Sections 4.1–4.3.
	23b	Discuss any limitations of the evidence included in the review.	Section 4.6.
	23c	Discuss any limitations of the review processes used.	Section 4.6 (lines 316–323).
	23d	Discuss implications of the results for practice, policy, and future research.	Sections 4.4 and 4.7.

PRISMA 2020 Checklist

Section and Topic	Item #	Checklist item	Location where item is reported
**OTHER INFORMATION**			
Registration and protocol	24a	Provide registration information for the review, including register name and registration number, or state that the review was not registered.	Section 2.1, PROSPERO CRD42025211876.
	24b	Indicate where the review protocol can be accessed, or state that a protocol was not prepared.	Section 2.1: protocol registered prospectively.
	24c	Describe and explain any amendments to information provided at registration or in the protocol.	None reported.
Support	25	Describe sources of financial or non-financial support for the review, and the role of the funders or sponsors in the review.	Section "Funding": Victor Babeș University of Medicine and Pharmacy Timișoara.
Competing interests	26	Declare any competing interests of review authors.	Declared: none.
Availability of data, code and other materials	27	Report which of the following are publicly available and where they can be found: template data collection forms; data extracted from included studies; data used for all analyses; analytic code; any other materials used in the review.	Section "Data Availability Statement": all data included in article; further inquiries to corresponding author.

From: Page MJ, McKenzie JE, Bossuyt PM, Boutron I, Hoffmann TC, Mulrow CD, et al. The PRISMA 2020 statement: an updated guideline for reporting systematic reviews. BMJ 2021;372:n71.doi: 10.1136/bmj.n71. This work is licensed under CC BY 4.0. To view a copy of this license, visit https://creativecommons.org/licenses/by/4.0/
